# Immunotherapy of Genitourinary Malignancies

**DOI:** 10.1155/2012/397267

**Published:** 2012-03-05

**Authors:** Teruo Inamoto, Haruhito Azuma

**Affiliations:** Department of Urology, Osaka Medical College, Takatsuki, Osaka 569-8686, Japan

## Abstract

Most cancer patients are treated with some combination of surgery, radiation, and chemotherapy. Despite recent advances in local therapy with curative intent, chemotherapeutic treatments for metastatic disease often remain unsatisfying due to severe side effects and incomplete long-term remission. Therefore, the evaluation of novel therapeutic options is of great interest. Conventional, along with newer treatment strategies target the immune system that suppresses genitourinary (GU) malignancies. Metastatic renal cell carcinoma and non-muscle-invasive bladder caner represent the most immune-responsive types of all human cancer. This review examines the rationale and emerging evidence supporting the anticancer activity of immunotherapy, against GU malignancies.

## 1. Introduction

The immune system is composed of two major subdivisions the innate immune system and the adaptive immune system. The innate immune system, comprised of cytokines, macrophages, and NK cells is rapidly responsive, while the adaptive system is antigen specific and relatively slow to develop.

 On the other hand, cellular immune responses including macrophages and T cell are involved in regulating malignancies. Immunotherapy using activated mononuclear cells is a way to harness the adaptive immune response, which is comprised of the antigen-presenting cells (APCs) including DCs and CD4^+^ and CD8^+^ T cells to fight malignancies. The APCs activate T cells by processing antigens and present them to T-cell receptors (TCRs) in the context of the MHC restriction, while CD4^+^ T cells include both helper and regulatory T cells (TREG).

 Humoral immune responses are usually thought to play an important role in inflammation, which is characterized by edema and the recruitment of phagocytic cells. Also, humoral immune responses including antibody are involved in regulating malignancies. Actually, these humoral factors are found in serum in patients with malignancies, or they are formed at the site of tumorigenesis.

 Immunological treatment strategies for cancer fall into two distinctive categories, namely, specific and nonspecific immunotherapy. Nonspecific immunotherapy induces inflammation or otherwise amplifies an already present immune response, for example, IFN, IL-2, and bacillus Calmette-Guerin (BCG). For decades urologists have successfully used nonspecific immunotherapy in the battle against cancer. BCG in non-muscle-invasive bladder cancer is standard primary therapy, and IL-2 in renal cell carcinoma is adjunctive therapy. In contrast, specific immunotherapy requires tumor-specific antigen recognition by T cells. Specific immunotherapy makes use of antigen-specific T lymphocytes or antibodies produced by B lymphocytes. Recently, prostate cancer vaccines have attempted to induce cancer-specific systemic immune responses and represent a new class of targeted therapies. Several immunotherapeutic strategies effective against prostate, bladder, or renal cancer in animal models are under clinical investigation for their efficacy against human GU malignancies. In addition to existing therapies, novel approaches that attempt to exploit the immune system ability to identify, target, and eradicate GU malignancies are now being developed. This review highlights current immunotherapy strategies that may prove to be successful treatments for GU malignancies.

## 2. Renal Cell Carcinoma

### 2.1. General Epidemiology and Disease Burden

Kidney cancer is the tenth leading cause of cancer deaths in males in the United States [1], and death rates of kidney cancer are the highest among American Indians/Alaskan Natives. In Europe approximately 14,000 people die annually of renal tumors with an estimated 27,000 new cases per year. About two-thirds of all patients present with localized disease, which can mostly be cured by radical or partial nephrectomy with a 60% to 70% 5-year survival rate. A third of patients present with metastatic disease and have a life expectancy of less than 12 months. The prognosis in patients with metastatic renal cell carcinoma (mRCC) is poor with approximately 1-year median survival and a 10% to 20% 2-year survival rate [2, 3]. This is largely due to the absence of effective chemotherapy agents and the limited usefulness of radiation therapy for mRCC.

### 2.2. Association with Obesity

Several risk factors for developing RCC have been reported, including smoking, hypertension, and obesity [4, 5]. The association to obesity is widely accepted and has been reported consistently in several studies [6–8]. There is evidence of elevated levels of numerous proinflammatory molecules in the blood of obese [9–11]. Also, the association between obesity and kidney disease is described, suggesting that inflammation could play an important role in the pathogenic mechanism of renal injury in obese patients [12, 13]. Indeed, C-reactive protein (CRP) represents a promising prognostic variable in patients with RCC [14–18].

### 2.3. Leptin and Its Role in RCC Development

Potential biologic mechanisms that have been hypothesized, including higher levels of estrogen and insulin, higher concentrations of growth factors in adipose tissue, and immune dysfunction [5]. Spyridopoulos et al. reported that levels of leptin, which is produced in adipose tissue and plays a modulatory role between metabolism and immunity, were inversely associated with RCC risk [19]. Adipose tissue produces a variety of inflammatory factors, including leptin, adiponectin, as well as cytokines. Indeed, plasma leptin levels are strongly associated with total adipose tissue mass [20–23]. The exact mechanism by which obesity, a state of chronic, slightly systemic inflammation, is a risk factor for developing RCC still remains unknown. Not only is plasma leptin increased in obese subjects, but leptin is decreased in adipose tissue mass induced by exercise [24] and anorexia nervosa [25]. Leptin also plays an antitumor effect through induction of NK cells proliferation and activation. Interestingly, it is reported that excess risk for developing RCC was observed among patients with low plasma levels of leptin, after adjusting for potential confounding factors, such as central obesity, DM and adiponectin [19]. Given the immunogenic nature of RCC and the role of leptin in the regulation of immunocompetence, crosstalk between lymphocytes and adipocytes may contribute to immune regulation in patients with RCC, contributing to tumor development.

### 2.4. Conventional Immunotherapy for Metastatic RCC

In addition to drugs already used in clinical practice, novel drugs are already under evaluation in clinical trials (Table 1). Immunotherapy for mRCC mainly involves the direct administration of effector molecules or cells to a patient and requires no relationship with the host immune system. Cytokines can indirectly affect tumor growth by inducing cytolytic T cells or by acting directly on tumor cells. Interferon (IFN)-alfa and IL-2 are widely studied examples of passive immunotherapy for mRCC. Since IL-2 has believed to have no direct impact on mRCC cells, the effect of IL-2 on mRCC is believed to be its ability to expand T-cell populations with antitumor activity [26]. If, however, the cytokine activate's the host immune system, these cytokine therapies are considered to be active immunotherapy. Evidence from knockout mice suggests that IL-2 is crucial for the homeostasis and function of CD4^+^ CD25^+^ regulatory T cells in vivo [27]. Although the response rate in patients with mRCC treated with IL-2 varies between 10% and 20%, some responses are durable. High-dose IL-2 appears to be able to cure a small percentage of highly selected patients [28].

### 2.5. Combination Immunotherapy

Some studies suggest that combination therapies involving IL-2 combined with additional cytokines may be more effective than IL-2 alone. Akaza et al. investigated the efficacy of combination therapy of low-dose IL-2 and IFN-alpha. In the 46 patients evaluated in Phase 1 and Phase 2, the response rate was 26.1% (12 of 46 patients), being highest in 38.7% (12 of 31 patients) of those who were nephrectomized, and with only lung metastases. Passalacqua et al. conducted Phase 3, randomised, multicentre trial of maintenance immunotherapy with low-dose IL-2 and IFN-alfa for mRCC patients [29]. Maintenance immunotherapy after disease progression was found to be feasible but did not significantly increase OS [29]. Cytokine therapy may be used in combination with chemotherapy as adjuvant therapy.

 Oblimersen is an 18-base oligodeoxynucleotide encoding antisense to the gene for bcl-2, an antiapoptotic protein that is upregulated in renal and other cancers. Margoline et al. evaluated the combination of oblimersen with IFN-alfa in mRCC [30]. They found that only 1 patient of 23 patients enrolled in the study had a partial response lasting 2.5 months, concluding oblimersen given in the dose and schedule used with IFN-alfa does not appear sufficiently active to warrant further study in mRCC [30].

### 2.6. Cytokines, Immunomodulators, and DCs

Another cytokine, IL-12, induces the differentiation of T cells into a T-helper-1(Th1) phenotype. CD8^+^ population of the Th1 phenotype is considered to be cytotoxic T lymphocyte (CTL) which is highly cytolytic. In a murine model combination therapy with IL-12 and IL-2 caused the regression of primary and metastatic disease with significantly better results than solo treatment with either agent. Granulocyte-macrophage colony-stimulating factor (GM-CSF) has received greater focus from the therapeutic perspective because of its ability to activate monocyte and macrophages, which can directly mediate antitumor activity through activated macrophages to release TNF-alfa. Garcia et al. conducted Phase 2 trial of subcutaneous IL-2, GM-CSF, and IFN-alfa in patients with mRCC [31]. The overall response rate was 20% (one complete response and 11 partial responses of 60 patients), and the median progression-free survival and overall survival were 6.0 and 23.4 months, respectively, suggesting the potent efficacy. GM-CSF also promotes dendritic cell (DC) differentiation and survival. Flt3 ligand (Flt3-L), a member of a growth factors that stimulate the proliferation of hematopoietic stem cells, is being tested for its ability to increase DC numbers [32]. Flt3 has also been tested in mRCC patients in clinical trials, and results demonstrated that although Flt3-L is capable of inducing the expansion of circulating DCs in patients with mRCC, it lacked relevant clinical activity at the doses and schedules examined [33, 34].

### 2.7. Tumor Vaccine

Active specific immunotherapy includes the vaccination of patients virtually to induce long-lasting, tumor-specific immunity which is capable of rejecting cancer cells as well as inducing safe and protective immunological memory. Autologous tumor cell vaccines are being used in an attempt to induce tumor-specific immune responses in mRCC. These vaccines include genetically altered tumor cells to enhance the immunogenicity of the tumor cells, thereby, inducing a tumor-specific T-cell response. Schendel et al. tested the possible application of vaccination with allogeneic tumor cells [35]. In the study, a human RCC cell line was genetically modified by retroviral transduction to express the costimulatory molecule CD80. It was possible to isolate CTL clones that were able to accomplish tumor lyses that expressed all of the corresponding allospecificities, demonstrating that the induction of allospecific responses did not hinder the development of tumor-associated CTLs in vitro. In practice, Lemoine et al. conducted Phase 2 trial to check whether systemic administration of IL-2 or infusion of DCs loaded with tumor extracts could lead to some response rates with concomitant survival improvements [36]. No adverse effect due to the vaccinations was observed in 5 patients enrolled. A specific immune response against autologous tumor cells was observed in the 2 of 5 patients who completed the treatment. A transient and massive increase of circulating natural regulatory T-cells was evidenced in 3 patients following IL-2 administration [36]. These results may support the use of modified allogeneic tumor cells for the vaccination of partial MHC-matched patients with RCC.

### 2.8. Molecular Targeted Therapy

Newer studies suggest that targeted therapy for mRCC, including sunitinib, sorafenib, temsirolimus, everolimus, or combined bevacizumab and IFN-alfa, has proved efficacious as first- or second-line treatment although they have not yet shown a statistically significant survival benefit except for temsirolimus in the high-risk mRCC subgroup. Thus, there is still an urgent need to improve pharmacotherapy for mRCC. Cho et al., conducted a retrospective analysis of the tolerability and efficacy of IL-2 therapy in patients who had previously received VEGF-targeted therapy including tyrosine-kinase inhibitor (TKI) [37]. No patients achieved a partial or complete response to therapy, and the incidence of severe cardiac toxicities in patients receiving prior TKI reached 40%, concluding that the assumption that IL-2 therapy can be safely administered after TKI therapy may not be valid [37]. Gollob et al. tested the activity and tolerability of TKI sorafenib administered with IFN-alpha-2b as first- or second-line therapy in mRCC patients [38]. In this Phase 2 study, the response rate was 33% (95% CI, 19% to 49%; 13 of 40 patients), including 28% partial responses (*n* = 11) and 5% complete responses (*n* = 2), with the median duration of response 12 months. Especially, responses were seen in treatment-naive and IL-2 treated patients [38]. Accumulating data by larger cohort will further the rationale for new drugs based on combination therapy with immunotherapy to enhance the effect of immunomodulators on patient survival.

### 2.9. Bladder Cancer

Bladder cancer is the 4th most common genitourinary cancer in men and the 7th in women with an incidence of more than 70,000 new cases in the United States in 2010 [1]. At presentation up roughly 80% of patients are found to have non-muscle-invasive bladder cancer (NMIBC), which is disease confined to the superficial layer of the bladder (mucosa, lamina propria), specifically Ta, T1 or carcinoma in situ (CIS) [39]. Approximately half of NMIBC is at risk for disease recurrence and progression. Therefore, they require adjuvant intravesical treatment after tumor resection. Currently the gold standard treatment for noninvasive bladder cancer is BCG instillation into the bladder, which is now the most commonly used intravesical treatment for high-risk NMIBC endorsed in European Association of Urology and American Urological Association practice guidelines [40–42]. Virtually it is believed that there exists a host-immune escape associated with bladder cancer. Hence, the generation of a localized immune response in the bladder by the intravesical administration of live BCG may transiently restore impaired immune system in the peritumoral bladder wall, so that BCG can elicit the tumor rejection. Some studies were conducted to evaluate the mechanism of binding of BCG within the bladder. In a mouse model, it is known that BCG attaches to the bladder wall only in areas with urothelial damage [43]. Studies performed using purified extracellular matrix proteins to identify the proteins responsible for attachment suggest that BCG preferentially attaches to surfaces coated with purified fibronectin (FN) and to a lesser extent to other purified proteins including laminin, collagen, or fibrinogen [43]. Immunological aspects of BCG therapy for NMIBC are related to the presence of delayed-type hypersensitivity. Inflammatory response occurs after BCG instillation in to the bladder, which is characterized by an induction of leukocyte subpopulations, such as granulocytes, CD4^+^ and CD8^+^ T cells, NK cells, B lymphocytes, activated lymphocytes, and dendritic cells. Following this cellular recruitment, cytokines characterized as part of the Th1 and Th2 immune response accumulate [44]. Cytokines which are in charge of the antitumor response are essentially those related to Th1, including IL-2, IL-12, TNF-alfa, and IFN-gamma [44]. Intravesical BCG, a nonspecific active immunotherapy, has been used in the intravesical treatment of NMIBC for about 35 years. Despite an initial treatment success many patients with NMIBC eventually have recurrence after intravesical BCG treatments. Patients with CIS are more at risk for recurrence or advanced disease. Despite BCG treatment 42% to 83% of patients with CIS associate with papillary NMIBC and 20% to 34% with primary CIS experience progression to muscle invasive disease [45]. Although BCG is an effective adjuvant treatment for preventing bladder cancer recurrence, it is associated with a high incidence of adverse effects, which include nausea, vomiting, weight loss, anorexia, bladder irritation, dysuria, polyuria, hematuria, cystitis, urinary urgency, urinary tract infections, flu-like syndrome, and so on [46, 47]. No consensus has been reached about the optimal dose for BCG therapy nor about how the toxicity of BCG treatment can be reduced. Variations in the reported frequency of BCG-associated adverse events could be caused by variations in the dose of BCG used. Therefore, dose reductions may be a potential option for the prevention of BCG-associated adverse events, particularly for those patients known to be intolerant to standard-dose BCG. Mack and Frick reported the results of a Phase 2 study with low-dose BCG therapy in high-risk NMIBC and concluded that low-dose BCG therapy is an effective treatment in high-risk T1 bladder cancer, especially with maintenance therapy to prevent progression and recurrence [48]. Long-term outcome of a low-dose intravesical BCG therapy for CIS of the bladder is reported [49]. Complete response was achieved in 84% of the patients, in whom the recurrence-free rate was 72.4% after 3 years and 61.9% after 5 years. The median CR duration was 37.5 months, suggesting the efficacy and safety of low-dose BCG therapy for CIS of the bladder. A number of unresolved questions surround the BCG host interplay which may be characterized by a number of different strains of BCG. BCG daughter strains are divided into the early strains: Japan, Birkhaug, Russia and Brazil, which were brought to each country between 1924 and 1926, and the late strains: Pasteur, Danish, Glaxo and Connaught, which were obtained after 1931. Although all of these strains are descendants of the original M. Bovis isolate, subsequent passage under different conditions has resulted in a variety of strains with unique genetic alterations. Differences of antitumor effect between the strains, if any, remain unknown. Only a few studies report the efficacy of different strain. Dutch South East Cooperative Urological Group evaluated BCG-Tice versus BCG-RIVM in 469 patients with pTa/pT1 carcinoma and found that there were no statistical differences were observed in toxicity between the two strains of BCG [50]. Connaught (Canada), Pasteur (France), Armand Frappier (Canada), and Tokyo 172 (Japan) were employed and studied for the efficacy in low-dose regimens [51–53]. Lamm reported that there were differences in complication rates with various BCG strains [47, 54]. The incidence of cystitis like symptoms, haematuria, and fever in our series was 64%, 40%, and 20%, respectively [47, 54]. Irie et al. prospectively evaluated the efficacy and adverse events of low dose (40 mg) Tokyo 172 strain. There was no significant difference in tumor recurrence rate between the low dose (40 mg) group and the standard dose (80 mg) group [55]. Similarly, Takashi et al. reported that the dose (40 versus 80 mg) of BCG was not a significant determinant for CR in patients with CIS of the bladder [56]. These studies suggest that 40 mg would be an adequate dose for Tokyo 172 strain, and comparable study is possible with a dose of 40 mg. No prospective studies have been conducted to compare low dose Tokyo 172 strain (40 mg) with other BCG substrains. Side effects are commonly manifested during BCG therapy. Delay or interruption of instillation due to side-effects may actually be detrimental to efficacy. So an important issue is whether a low-dose regimen can reduce toxicity while maintaining efficacy. From a Phase III randomized trial comparing low-dose versus standard-dose BCG (Pasteur strain, 75 versus 150 mg), Pagano et al. reported a significant decrease in most of the common side effects (cystitis, fever, haematuria; *P* < 0.05), clarifying the relationships between dose and toxicity [52]. Studies, however, provided only short-term followup ranging from months to two years and data on the long-term condition of bladder patients originally treated with low-dose BCG is very rare. Losa et al. retrospectively reviewed 70 consecutive patients with primary or secondary carcinoma in situ with or without concomitant solitary or multifocal papillary tumor treated with weekly instillations of low-dose Pasteur strain for 6 weeks with median followup of 74 months [51]. Mean time was 18 months (range 6 to 69) to treatment failure and 13 months (range 7 to 53) to progression [51]. They conclude that low-dose BCG is similarly effective, with a lower incidence of side effects and long-lasting positive outcome. Similarly, Kamel et al. retrospectively evaluated 74 patients with G3, T1 bladder cancer treated by a 6-week course of low-dose Pasteur strain with median followup of 61 months [57]. Median time to treatment failure was 20 months. Regarding toxicity, irritative symptoms occurred in 24% of patients, fever in 9%, microscopic hematuria in 14%, which appeared to be lower when compared with the rates reported for regular doses of BCG [57].

 Usage of low-dose BCG for aggressive bladder cancer is controversial. Hurle et al. assessed the effectiveness of low-dose BCG for high-risk T1/G3 bladder cancer patients who had weekly instillations of low-dose Pasteur strain for 6-week [58]. With a median followup of 33 months, 28 of 51 patients (54.9%) were disease-free, and the risk of treatment failure was significantly greater for solid than papillary tumors (*P* = 0.0006), recurrent than primary tumors (*P* = 0.0052), and coexisting carcinoma in situ (*P* = 0.124) in multivariate analysis, suggesting that this low-dose Pasteur BCG is effective in the treatment of high-risk NMIBC, except for some tumor characteristics, such as solid appearance, coexisting carcinoma in situ, history of superficial transitional cell carcinoma, and early relapse after the initial induction course [58]. In contrast, Herr concluded that patients with highly malignant bladder cancer would not benefit from a dose reduction [59]. Now maintenance BCG therapy is proved to be useful for high-risk NMIBC patients. Although, long-term tolerance remains an important issue with maintenance schedule. Because complex immunological pathway contributes to the success of BCG therapy, stratification of the patients by their immunological aspects may help physicians to predict the response to BCG.

## 3. Prostate Cancer

Immunotherapy has also been carried out on other GU malignancies, except for mRCC and NMIBC, and primarily the prostate cancer has been a particular focus of immunotherapy. Despite improvements in surgery or radiotherapy for localized prostate cancer, 30% will develop metastatic disease. Once become metastatic, usually to the bone, the disease is no longer curable and is usually treated with androgen depletion therapy (ADT). Some studies support the evidenced that ADT induce an immune response against the prostate, which includes T-cell infiltration of the prostate and induce thymic regeneration. Disappointingly, over 50% of these patients treated by ADT progress to castration-resistant prostate cancer (CRPC) within a median of 18 to 24 months. CRPC remains an incurable disease when treated with docetaxel-based chemotherapy, and prednisone currently the only FDA-approved chemotherapeutic agent for the treatment of CRPC, because docetaxel-prednisone extended median overall survival modestly to 19 months and only 20% patients attained 3-year survival based on the results of two large randomized trials [60, 61]. Several immunotherapeutic strategies effective against prostate cancer in animal models are under clinical investigation for their efficacy against human CRPC. Cytokines, including IL-2 and IFN-alfa, which are mainly used in mRCC patients may also be useful for treating other GU malignancies. In a pilot study of CRPC involving IL-2 and IFN-alfa administration, some partial responses and PSA serum level decreases were reported. In the last 10 years, many cancer vaccines, to be specific active immunotherapy against specific tumor-associated antigens, were tried in clinical trials. These vaccines include DC, whole tumor cell, peptide, viral vectors, and so on. Although most of these vaccines were not approved by the US FDA. Most recently, US FDA approval of 2 immunotherapies including sipuleucel-T (Provenge, Dendreon Corp, WA, USA) and ipilimumab (Yervoy, Bristol-Myers Squibb) [62]. Sipuleucel-T is a novel autologous dendritic cell-based vaccine, and the tissue-specific antigen for immunization is prostatic acid phosphatase (PAP), which is expressed in about 95% of prostate tumors, and has highly specific expression for prostatic tissue. PAP is linked to GM-CSF, so that GM-CSF functions to enable efficient GM-CSF-receptor-mediated uptake of the PAP antigen, resulting in enhancing its antigenicity and DC-stimulating properties, moiety and augments the immune response. In a randomized crossover trial, 127 previously untreated men with metastatic CRPC were assigned [63]. The median survival for sipuleucel-T was significantly better than for placebo (25.9 versus 21.4 months, *P* = 0.01) and 3-year survival was prolonged with sipuleucel-T (34 versus 11%, *P* = 0.0046). Also, in the double-blind, placebo-controlled, multicenter Phase 3 trial, there was a relative reduction of 22% in the risk of death in the sipuleucel-T group as compared with the placebo group (*P* = 0.03), suggesting that sipuleucel-T prolongs overall survival among men with metastatic CRPC [64]. The activation of negative regulatory signals in T cells is required to avert an unduly exuberant immune response, which include cytotoxic T-lymphocyte antigen-4 (CTLA-4) which is essential for maintaining tolerance for self-antigens. Ipilimumab, CTLA-4-inhibiting fully human monoclonal antibodies have demonstrated objective clinical and PSA responses in advanced CRPC in Phase 1 and Phase 2 trials. In addition, other novel targets for immunotherapy against CRPC are likely to expand the therapeutic venue in the near future. Therefore, the proper sequence of immunotherapy and appropriate selection of patients will assume benefit.

## 4. Conclusion

Recent advances in immunotherapy in GU malignancies have provided insight into the complexity of immune manipulation and the promise of improving efficacy and reducing adverse reactions by better understanding of mechanisms of immunotherapy. Despite the clinical failures of some immunological treatments, several therapies do warrant further exploration.

## Figures and Tables

**Table 1:** Summary of immunotherapies and targeted agents in mRCC.

Agent	Class	Mechanism of action	Stage of treatment	PFS (months)
IL-2	Cytokine	Modulation of the host's immune response	First line for selected patients	
IFN-alfa	Cytokine	Activate NK cells and macrophages	Second line	3.1–5.4
IFN-alfa + IL-2	Cytokine	Modulation of the host's immune response	First line for selected patients	
IL-12	Cytokine	Modulation of the host's immune response	Phase II	
IL-2 + GM-CSF + IFN-alfa	Cytokine	Modulation of the host's immune response	Phase II	6
Sunitinib	Small molecule	TKI of VEGFR, PDGFR	First line/second line	11.1
Sorafenib	Small molecule	TKI of VEGFR, PDGFR, Ras	First line/second line	5.5
Temsirolimus	Small molecule	mTOR inhibitor	First line (used for poor risk)	5.5
Everolimus	Small molecule	mTOR inhibitor	Second line (used for TKI/IFN refractory mRCC)	
Pazopanib	Small molecule	TKI of VEGFR, PDGFR	Phase II, III	
Vatalanib	Small molecule	TKI of VEGFR, PDGFR	Phase I	
Imatinib	Small molecule	TKI of PDGR	Phase II	
Gefitinib	Small molecule	TKI of EGFR	Phase II	
Erlotinib	Small molecule	TKI of EGFR	Phase II	
Bortezomib	Small molecule	26 S proteasome inhibitor	Phase II	
Lapatinib	Small molecule	TKI of EGFR/Erb/2	Phase II, III	
Oblimersen	Oligo	Antisense oligo	Phase II	
Tumor vaccination	Protein	Tumor-specific T-cell response	Phase II	
Bevacizumab (+ IFN-alfa)	Mab	Antibody to VEGF	First line	8.5–10.2
Cetuximab	Mab	Antibody to EGFR	Phase II	
VEGF-trap	Mab	Antibody to VEGF	Phase I, II	
G250	Mab	Antibody to CA IX	Phase II	

Abbreviations: TKI; tyrosine kinase inhibitor, IFN; interferon, mRCC; metastatic renal cell carcinoma, NK; natural killer, Mab; monoclonal antibody.

## References

[1] Jemal A, Siegel R, Xu J, Ward E (2010). Cancer statistics, 2010. *CA: Cancer Journal for Clinicians*.

[2] Naito S, Yamamoto N, Takayama T (2010). Prognosis of Japanese metastatic renal cell carcinoma patients in the cytokine era: a cooperative group report of 1463 patients. *European Urology*.

[3] Pizza G, De Vinci C, Conte GL (2001). Immunotherapy of metastatic kidney cancer. *International Journal of Cancer*.

[4] Donat SM, Salzhauer EW, Mitra N, Yanke BV, Snyder ME, Russo P (2006). Impact of body mass index on survival of patients with surgically treated renal cell carcinoma. *Journal of Urology*.

[5] Moyad MA (2001). Obesity, interrelated mechanisms, and exposures and kidney cancer. *Seminars in Urologic Oncology*.

[6] Arends J (2010). Metabolism in cancer patients. *Anticancer Research*.

[7] Bauer F, Elbers CC, Adan RAH (2009). Obesity genes identified in genome-wide association studies are associated with adiposity measures and potentially with nutrient-specific food preference. *The American Journal of Clinical Nutrition*.

[8] Bingham S, Luben R, Welch A, Tasevska N, Wareham N, Khaw KT (2007). Epidemiologic assessment of sugars consumption using biomarkers: comparisons of obese and nonobese individuals in the European prospective investigation of cancer Norfolk. *Cancer Epidemiology Biomarkers and Prevention*.

[9] Civera M, Urios A, Garcia-Torres ML (2010). Relationship between insulin resistance, inflammation and liver cell apoptosis in patients with severe obesity. *Diabetes/Metabolism Research and Reviews*.

[10] García-Lafuente A, Guillamón E, Villares A, Rostagno MA, Martínez JA (2009). Flavonoids as anti-inflammatory agents: implications in cancer and cardiovascular disease. *Inflammation Research*.

[11] Rosado JO, Salvador M, Bonatto D (2007). Importance of the trans-sulfuration pathway in cancer prevention and promotion. *Molecular and Cellular Biochemistry*.

[12] Lee SW, Park GH, Lee SY, Song JH, Kim MJ (2005). Comparison of anthropometric data between end-stage renal disease patients undergoing hemodialysis and healthy adults in Korea. *Yonsei Medical Journal*.

[13] Navarro-Díaz M, Serra A, López D, Granada M, Bayés B, Romero R (2008). Obesity, inflammation, and kidney disease. *Kidney International*.

[14] Zurita AJ, Jonasch E, Wang X (2012). A cytokine and angiogenic factor (CAF) analysis in plasma for selection of sorafenib therapy in patients with metastatic renal cell carcinoma. *Annals of Oncology*.

[15] Zhu Z, Zhong S, Shen Z (2011). Targeting the inflammatory pathways to enhance chemotherapy of cancer. *Cancer Biology & Therapy*.

[16] Zhang X, Shi X, Li J (2011). Novel immunotherapy for metastatic bladder cancer using vaccine of human interleukin-2 surface-modified MB 49 cells. *Urology*.

[17] Zarour HM, Ferrone S (2011). Cancer immunotherapy: progress and challenges in the clinical setting. *European Journal of Immunology*.

[18] Yates DR, Rouprêt M (2011). Contemporary management of patients with high-risk non-muscle-invasive bladder cancer who fail intravesical BCG therapy. *World Journal of Urology*.

[19] Spyridopoulos TN, Petridou ET, Dessypris N (2009). Inverse association of leptin levels with renal cell carcinoma: results from a case-control study. *Hormones*.

[20] Drabkin HA, Gemmill RM (2010). Obesity, cholesterol, and clear-cell renal cell carcinoma (RCC). *Advances in Cancer Research*.

[21] Martin SS, Qasim A, Reilly MP (2008). Leptin resistance: a possible interface of inflammation and metabolism in obesity-related cardiovascular disease. *Journal of the American College of Cardiology*.

[22] Johnston A, Arnadottir S, Gudjonsson JE (2008). Obesity in psoriasis: leptin and resistin as mediators of cutaneous inflammation. *British Journal of Dermatology*.

[23] Hu X, Juneja SC, Maihle NJ, Cleary MP (2002). Leptin—a growth factor in normal and malignant breast cells and for normal mammary gland development. *Journal of the National Cancer Institute*.

[24] Hickey MS, Considine RV, Israel RG (1996). Leptin is related to body fat content in male distance runners. *American Journal of Physiology*.

[25] Kumar KK, Tung S, Iqbal J (2010). Bone loss in anorexia nervosa: leptin, serotonin, and the sympathetic nervous system. *Annals of the New York Academy of Sciences*.

[26] Mulder SF, Jacobs JFM, Olde Nordkamp MAM (2011). Cancer patients treated with sunitinib or sorafenib have sufficient antibody and cellular immune responses to warrant influenza vaccination. *Clinical Cancer Research*.

[27] Ahmadzadeh M, Rosenberg SA (2006). IL-2 administration increases CD4^+^CD25^hi^ Foxp3^+^ regulatory T cells in cancer patients. *Blood*.

[28] Coppin C (2008). Immunotherapy for renal cell cancer in the era of targeted therapy. *Expert Review of Anticancer Therapy*.

[29] Passalacqua R, Buzio C, Buti S (2010). Phase III, randomised, multicentre trial of maintenance immunotherapy with low-dose interleukin-2 and interferon-*α* for metastatic renal cell cancer. *Cancer Immunology, Immunotherapy*.

[30] Margolin K, Synold TW, Lara P (2007). Oblimersen and *α*-interferon in metastatic renal cancer: a phase II study of the California Cancer Consortium. *Journal of Cancer Research and Clinical Oncology*.

[31] Garcia JA, Mekhail T, Elson P (2012). Phase I/II trial of subcutaneous interleukin-2, granulocyte-macrophage colony-stimulating factor and interferon-*α* in patients with metastatic renal cell carcinoma. *BJU International*.

[32] Somers KD, Brown RR, Holterman DA (2003). Orthotopic treatment model of prostate cancer and metastasis in the immunocompetent mouse: efficacy of flt3 ligand immunotherapy. *International Journal of Cancer*.

[33] Morelli AE, Coates PTH, Shufesky WJ (2007). Growth factor-induced mobilization of dendritic cells in kidney and liver of rhesus macaques: implications for transplantation. *Transplantation*.

[34] Lierman E, Lahortiga I, Van Miegroet H, Mentens N, Marynen P, Cools J (2007). The ability of sorafenib to inhibit oncogenic PDGFR*β* and FLT3 mutants and overcome resistance to other small molecule inhibitors. *Haematologica*.

[35] Schendel DJ, Frankenberger B, Jantzer P (2000). Expression of B7.1 (CD80) in a renal cell carcinoma line allows expansion of tumor-associated cytotoxic T lymphocytes in the presence of an alloresponse. *Gene Therapy*.

[36] Lemoine FM, Cherai M, Giverne C (2009). Massive expansion of regulatory T-cells following interleukin 2 treatment during a phase I-II dendritic cell-based immunotherapy of metastatic renal cancer. *International Journal of Oncology*.

[37] Cho DC, Puzanov I, Regan MM (2009). Retrospective analysis of the safety and efficacy of interleukin-2 after prior VEGF-targeted therapy in patients with advanced renal cell carcinoma. *Journal of Immunotherapy*.

[38] Gollob JA, Rathmell WK, Richmond TM (2007). Phase II trial of sorafenib plus interferon alfa-2b as first- or second-line therapy in patients with metastatic renal cell cancer. *Journal of Clinical Oncology*.

[39] Boorjian SA, Zhu F, Herr HW (2010). The effect of gender on response to bacillus Calmette-Guérin therapy for patients with non-muscle-invasive urothelial carcinoma of the bladder. *BJU International*.

[40] Babjuk M, Oosterlinck W, Sylvester R (2011). EAU guidelines on non-muscle-invasive urothelial carcinoma of the bladder, the 2011 update. *European Urology*.

[41] Stenzl A, Cowan NC, De Santis M (2009). The updated EAU guidelines on muscle-invasive and metastatic bladder cancer. *European Urology*.

[42] Hall MC, Chang SS, Dalbagni G (2007). Guideline for the management of nonmuscle invasive bladder cancer (stages Ta, T1, and Tis): 2007 update. *Journal of Urology*.

[43] Ratliffe TL, Kavoussi LR, Catalona WJ (1988). Role of fibronectin in intravesical BCG therapy for superficial bladder cancer. *Journal of Urology*.

[44] Chade DC, Borra RC, Nascimento IP (2008). Immunomodulatory effects of recombinant BCG expressing pertussis toxin on TNF-alpha and IL-10 in a bladder cancer model. *Journal of Experimental and Clinical Cancer Research*.

[45] Lamm DL, Blumenstein BA, Crissman JD (2000). Maintenance bacillus Calmette-Guérin immunotherapy for recurrent TA, T1 and carcinoma in situ transitional cell carcinoma of the bladder: a randomized Southwest Oncology Group Study. *Journal of Urology*.

[46] Wishahi MM, Ismail IMH, El-Sherbini M (1994). Immunotherapy with bacille Calmette-Guérin in patients with superficial transitional cell carcinoma of the bladder associated with bilharziasis. *British Journal of Urology*.

[47] Lamm DL, van der Meijden APM, Morales A (1992). Incidence and treatment of complications of bacillus Calmette-Guérin intravesical therapy in superficial bladder cancer. *Journal of Urology*.

[48] Mack D, Frick J (1995). Low-dose bacille Calmette-Guérin (BCG) therapy in superficial high-risk bladder cancer: a phase II study with the BCG strain Connaught Canada. *British Journal of Urology*.

[49] Mugiya S, Ozono S, Nagata M (2005). Long-term outcome of a low-dose intravesical bacillus Calmette-Guérin therapy for carcinoma *in situ* of the bladder: results after six successive instillations of 40 mg BCG. *Japanese Journal of Clinical Oncology*.

[50] Witjes WPJ, Witjes JA, Oosterhof GON, Debruyne FMJ (1996). Update on the Dutch Cooperative Trial: mitomycin versus bacillus Calmette-Guérin-Tice versus bacillus Calmette-Guérin RIVM in the treatment of patients with pTA-pT1 papillary carcinoma and carcinoma in situ of the urinary bladder. Dutch South East Cooperative Urological Group. *Seminars in Urologic Oncology*.

[51] Losa A, Hurle R, Lembo A (2000). Low dose bacillus Calmette-Guérin for carcinoma in situ of the bladder: long-term results. *Journal of Urology*.

[52] Pagano F, Bassi P, Piazza N, Abatangelo G, Drago Ferrante GL, Milani C (1995). Improving the efficacy of BCG immunotherapy by dose reduction. *European Urology*.

[53] Pagano F, Bassi P, Milani C, Meneghini A, Maruzzi D, Garbeglio A (1991). A low dose bacillus Calmette-Guérin regimen in superficial bladder cancer therapy: is it effective?. *Journal of Urology*.

[54] Lamm DL (1992). Optimal BCG treatment of superficial bladder cancer as defined by American trials. *European Urology*.

[55] Irie A, Uchida T, Yamashita H (2003). Sufficient prophylactic efficacy with minor adverse effects by intravesical instillation of low-dose bacillus Calmette-Guérin for superficial bladder cancer recurrence. *International Journal of Urology*.

[56] Takashi M, Wakai K, Ohno Y, Murase T, Miyake K (1995). Evaluation of a low-dose intravesical bacillus Calmette-Guérin (Tokyo strain) therapy for superficial bladder cancer. *International Urology and Nephrology*.

[57] Kamel AI, El Baz AG, Abdel Salam WT, El Din Ryad ME, Mahena AA (2009). Low dose BCG regimen in T1 transitional cell carcinoma of the bladder: long term results. *Journal of the Egyptian National Cancer Institute*.

[58] Hurle R, Losa A, Ranieri A, Graziotti P, Lembo A (1996). Low dose Pasteur bacillus Calmette-Guérin regimen in stage T1, grade 3 bladder cancer therapy. *Journal of Urology*.

[59] Herr HW, Schwalb DM, Zhang ZF (1995). Intravesical bacillus Calmette-Guérin therapy prevents tumor progression and death from superficial bladder cancer: ten-year follow-up of a prospective randomized trial. *Journal of Clinical Oncology*.

[60] Petrylak DP, Tangen CM, Hussain MHA (2004). Docetaxel and estramustine compared with mitoxantrone and prednisone for advanced refractory prostate cancer. *The New England Journal of Medicine*.

[61] Tannock IF, de Wit R, Berry WR (2004). Docetaxel plus prednisone or mitoxantrone plus prednisone for advanced prostate cancer. *The New England Journal of Medicine*.

[62] Sonpavde G, Agarwal N, Choueiri TK, Kantoff PW (2011). Recent advances in immunotherapy for the treatment of prostate cancer. *Expert Opinion on Biological Therapy*.

[63] Small EJ, Schellhammer PF, Higano CS (2006). Placebo-controlled phase III trial of immunologic therapy with Sipuleucel-T (APC8015) in patients with metastatic, asymptomatic hormone refractory prostate cancer. *Journal of Clinical Oncology*.

[64] Kantoff PW, Higano CS, Shore ND (2010). Sipuleucel-T immunotherapy for castration-resistant prostate cancer. *The New England Journal of Medicine*.

